# Toxic side-effects of diaspirin cross-linked human hemoglobin are attenuated by the apohemoglobin-haptoglobin complex

**DOI:** 10.1016/j.biopha.2024.116569

**Published:** 2024-04-10

**Authors:** Carlos J. Munoz, Daniela Lucas, Jacinda Martinez, Mia Ricario, Quintin T. O’Boyle, Ivan S. Pires, Andre F. Palmer, Pedro Cabrales

**Affiliations:** a Department of Bioengineering, University of California San Diego, La Jolla, CA, United States; b William G. Lowrie Department of Chemical and Biomolecular Engineering, The Ohio State University, Columbus, OH, United States

**Keywords:** Hemoglobin, Heme, Scavenging, Microcirculation, Microhemodynamics, HBOC

## Abstract

Alpha-alpha diaspirin-crosslinked human hemoglobin (DCLHb or ααHb) was a promising early generation red blood cell (RBC) substitute. The DCLHb was developed through a collaborative effort between the United States Army and Baxter Healthcare. The core design feature underlying its development was chemical stabilization of the tetrameric structure of hemoglobin (Hb) to prevent Hb intravascular dimerization and extravasation. DCLHb was developed to resuscitate warfighters on the battlefield, who suffered from life-threatening blood loss. However, extensive research revealed toxic side effects associated with the use of DCLHb that contributed to high mortality rates in clinical trials. This study explores whether scavenging Hb and heme via the apohemoglobin-haptoglobin (apoHb-Hp) complex can reduce DCLHb associated toxicity. Awake Golden Syrian hamsters were equipped with a window chamber model to characterize the microcirculation. Each group was first infused with either Lactated Ringer’s or apoHb-Hp followed by a hypovolemic infusion of 10% of the animal’s blood volume of DCLHb. Our results indicated that animals pretreated with apoHb-Hb exhibited improved microhemodynamics vs the group pretreated with Lactated Ringer’s. While systemic acute inflammation was observed regardless of the treatment group, apoHb-Hp pretreatment lessened those effects with a marked reduction in IL-6 levels in the heart and kidneys compared to the control group. Taken together, this study demonstrated that utilizing a Hb and heme scavenger protein complex significantly reduces the microvasculature effects of ααHb, paving the way for improved HBOC formulations. Future apoHb-Hp dose optimization studies may identify a dose that can completely neutralize DCLHb toxicity.

## 1. Introduction

Blood transfusions are vital in clinical medicine, serving as the primary treatment for numerous diseases such as sickle cell anemia, thalassemia, and trauma. However, challenges such as the quality of stored blood and donor scarcity have fueled research into the development of blood alternatives [1–3]. One promising avenue is the development of hemoglobin-based oxygen carriers (HBOCs) to serve as oxygen carriers or red blood cell (RBC) substitutes for use in tissue reperfusion and oxygen delivery [3]. Unfortunately, no research group has successfully produced an HBOC without adverse side-effects [2]. One HBOC, alpha-alpha diaspirin cross-linked hemoglobin (ααHb or DCLHb), created through a collaboration between the United States Army and Baxter Pharmaceuticals, showed great promise [4–6]. This product cross-linked the two alpha (α) subunits of the hemoglobin (Hb) tetramer to prevent its dimerization into smaller subunits, in order to prevent extravasation of αβ dimers into tissues and potential downstream side-effects [6–9].

Regrettably, ααHb failed during phase III clinical trials, demonstrating a higher mortality rate than the control group during hemorrhagic shock [10]. The increase in blood pressure (BP) observed with ααHb was attributed to its ability to scavenge nitric oxide (NO), a known gaseous vasorelaxation signal in smooth muscle, and Hb extravasation into the tissue space [11,12]. Unfortunately, ααHb was not the only commercial HBOC to face such challenges. HBOCs produced by Biopure, Northfield Laboratories, Sangart, and HemoBioTech encountered similar issues and met similar fates [13].

To address the side-effects associated with acellular HBOCs, we propose harnessing naturally occurring Hb and heme detoxification mechanisms to mitigate vasoconstriction, systemic hypertension, and oxidative tissue injury. The naturally occurring Hb-scavenging plasma protein, haptoglobin (Hp), plays a pivotal role in detoxifying cell-free Hb in the blood by binding to it and facilitating its clearance via CD163-mediated endocytosis, which neutralizes the side-effects of cell-free Hb [14]. Previous studies have shown that Hp administration can normalize vascular NO signaling following hemolysis [15,16].

Hemopexin (Hpx) is another plasma protein, which scavenges heme released from cell-free Hb after Hb auto-oxidation into methemoglobin [17,18]. By compartmentalizing heme within the Hpx-heme complex, heme is prevented from catalyzing oxidative reactions with blood and tissue components, thus averting lipid, protein, and nucleic acid oxidation [19,20]. A potential cost-effective alternative to Hpx is heme-free apohemoglobin (apoHb). This molecule can scavenge free heme due to its specificity for heme and the highly hydrophobic nature of its vacant heme-binding pocket [21,22]. Our group has demonstrated that it is possible to react apoHb with Hp to yield the protein complex (apoHb-Hp), which is capable of scavenging both Hb and heme in plasma in an exogeneous Hb challenge model [15]. We have further demonstrated that the apoHb-Hp complex can limit the side-effects associated with cell-free Hb in sickle cell anemia and beta-thalassemia [23,24]. More germane to this study, we also demonstrated that the apoHb-Hp complex can limit the side-effects associated with administration low molecular weight polymerized hemoglobin molecules [25].

In this study, we hypothesize that the apoHb-Hp complex can attenuate the side-effects associated with the administration of ααHb, which should improve the performance of the HBOC by improving microhemodynamics and inflammation in vital organs. To address this question, systemic parameters and microvascular function were evaluated in Golden Syrian hamsters pretreated with either apoHb-Hp or lactated Ringer’s (control group) prior to 10% hypovolemic infusion with ααHb. Hamsters were instrumented with a dorsal window chamber, and a catheter in the left common carotid artery. Microvascular perfusion was characterized by measuring blood vessel diameter, blood flow rate, and functional capillary density. Systemic variables, such as mean arterial pressure (MAP) and heart rate (HR) were also monitored. A histology report was generated to assess toxicity in the spleen, kidney, liver, and heart.

## 2. Methods

### 2.1. ApoHb and Hp preparation

The apoHb used in this study was prepared from human Hb via tangential flow filtration based on the acidic-ethanol heme-extraction procedure as previously described in the literature [22]. The absence of residual heme was verified by measuring the ratio of absorbance between the Soret peak (λ_max_ = 412–413 nm) and 280 nm to ensure less than 1% residual heme in the apoHb product. The heme-binding capacity of apoHb preparations was 80% [21]. Human Hp was purified from human Cohn fraction IV (FIV) purchased from Seraplex (Pasadena, CA) as described in the literature [26]. The final Hp protein solution was composed of a mixture of Hp2–1 and Hp2–2 polymers with an average moelcular weight (MW) of 400–500 kDa. The apoHb-Hp complex was formed by reacting apoHb with Hp, and complex formation was confirmed by analysis of the mixture via size exclusion HPLC (HPLC-SEC). A more descriptive explanation was published elsewhere [15].

### 2.2. DCLHb (ααHb) preparation

The ααHb used followed similar methods of production. Briefly, DCLHb was prepared by reacting deoxygenated human Hb with bis(3,5-dibromosalicyl) fumarate, as previously described [27]. This reaction took place in a 0.2 M bis-Tris buffer at a pH of 7.2, at 37 °C for 2 hours, with both Hb and the cross-linking reagent at a concentration of 1 mM each. The process was carried out anaerobically. After the reaction, glycine was added to neutralize any remaining reagent, and no measures were taken to exclude oxygen post this addition. The reaction mixture was then dialyzed against a 0.2 M glycine buffer (pH 8.0), followed by chromatography on a DEAE-cellulose column with a NaCl gradient for elution. Optical density monitoring at 540 nm helped identify the elution profile, revealing unmodified Hb as the largest fraction and the first modified peak representing Hb cross-linked between the α chains. Further analysis of these fractions was conducted through isoelectric focusing and two-dimensional gel electrophoresis. The DCLHb was kept at −80 °C until time of use and thawed carefully by gradually raising the temperature over a 24-h period prior to infusion.

### 2.3. Animal preparation

**Fig. 1.**
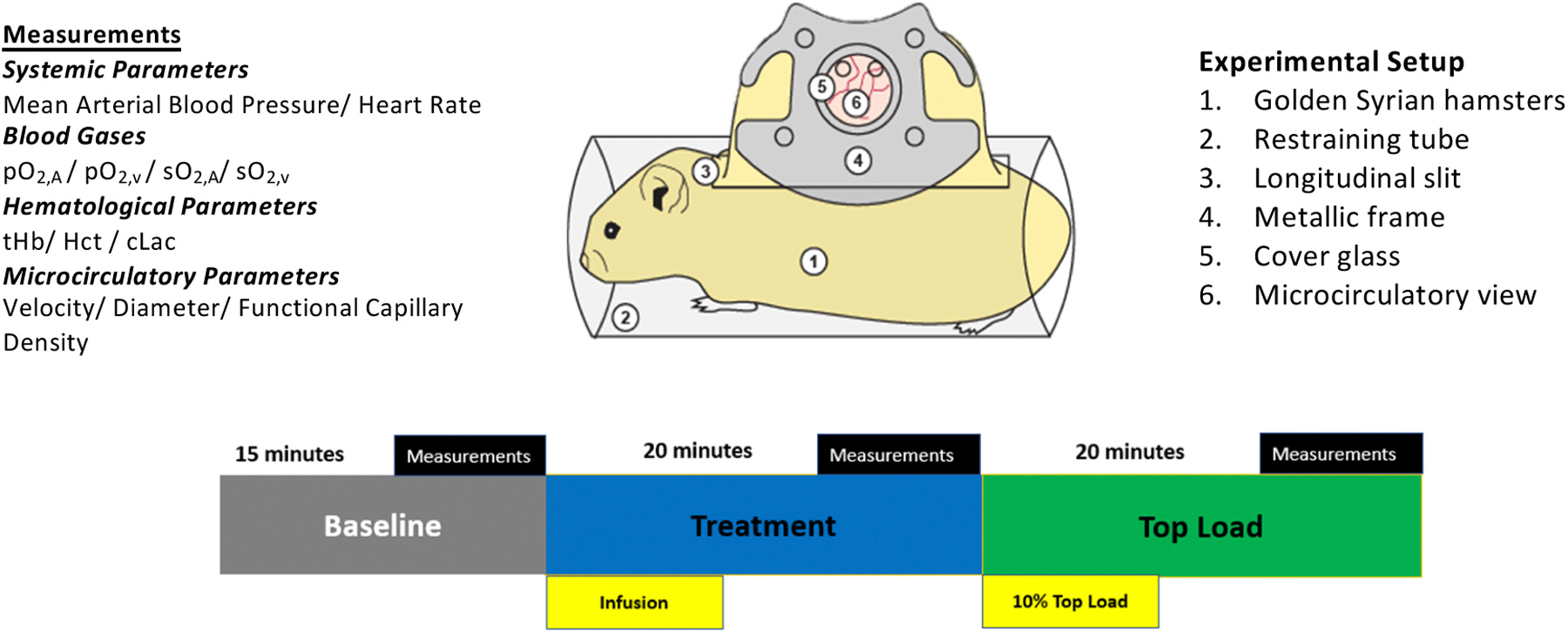
Experimental model and timeline. Animals were subjected to a three-stage experimental process consisting of baseline assessment, administration of a treatment solution and assessment, followed by administration of ααHb and assessment. Baseline microcirculatory and systemic parameters were measured, and the treatment group was administered an infusion of either lactated Ringer’s solution or apoHb-Hp complex where the same parameters measured during the baseline phase were assessed after 20 min, and finally the experimental group was administered an infusion of ααHb followed after 20 min by measuring the same parameters during the treatment phase.

Investigations were performed in 55–65 g male Golden Syrian hamsters (Charles River Laboratories, Boston, MA) fitted with a dorsal skinfold window chamber. Animal handling and care followed the NIH Guide for the Care and Use of Laboratory Animals. The experimental protocol was approved by the local animal care committee. The hamster window chamber model is widely used for microvascular studies in the unanesthetized state, and the complete surgical technique is described in detail elsewhere [27,28]. The experimental animal was allowed at least 2 days to recover before the preparation was assessed under the microscope for any signs of edema, bleeding, or unusual neovascularization. Animals were anesthetized again, and arterial and venous catheters filled with a heparinized saline solution (30 IU/mL) were implanted. Catheters were tunneled under the skin, exteriorized at the dorsal side of the neck, and securely attached to the window frame. The microvasculature was examined 3–4 days after the initial surgery and only animals with window chambers whose tissue did not present regions of low perfusion, inflammation, and edema were entered into the study [29]. The animal preparation is illustrated in Fig. 1.

### 2.4. Inclusion criteria

Animals were suitable for the experiments if 1) systemic variables were within a normal range, namely, heart rate (HR) > 340 beat/min, mean arterial blood pressure (MAP) > 80 mmHg, systemic hematocrit (Hct) > 45%, and arterial oxygen partial pressure (PaO_2_) > 50 mmHg; and 2) microscopic examination of the tissue in the chamber observed under a 650 × magnification did not reveal signs of edema or bleeding. Hamsters are a fossorial species with a lower arterial PO_2_ than other rodents due to their adaptation to a subterranean environment. However, microvascular PO_2_ distribution in the chamber window model is the same as in other rodents such as mice [30].

### 2.5. Hypervolemic infusion (top-load) protocol

Experimental groups were labeled based on the infusion of 0.63 mL of the treatment solution, which consisted of either the control (lactated Ringer’s solution) or apoHb-Hp. The concentration of the apoHb-Hp solution was 62 mg/mL, with an average dose of 550 mg/kg per animal. The treated animals were then challenged with ααHb [6] at a concentration of 50 mg/mL at an average dose of 300 mg/kg per animal, which was administered as a bolus through the carotid artery. After each infusion, animals were allowed 15–20 min to stabilize before systemic and microvascular characterization. The experimental timeline is described in Fig. 1.

### 2.6. Systemic variables and blood chemistry

The arterial cannula was connected to a pressure transducer and recording system (MP150, Biopac, Santa Barbara, CA), and blood pressure signals was recorded, along with MAP, and HR. The Hct was measured from centrifuged arterial blood samples taken in heparinized capillary tubes. Hb content was determined spectrophotometrically from a single drop of blood (B-Hemoglobin, Hemocue, Stockholm, Sweden). Arterial blood was collected in heparinized glass capillaries (50 μL) and immediately analyzed for pO_2_, pCO_2_, pH, electrolytes, lactate, and total Hb (tHb) content (ABL90; Radiometer America, Brea, CA).

### 2.7. Microvascular experimental setup

The unanesthetized animal was placed in a restraining tube with a longitudinal slit from which the window chamber protruded, then fixed to the microscopic stage of a transillumination intravital microscope (BX51WI, Olympus, New Hyde Park, NY). The animals were given 20 min to adjust to the change in the tube environment before measurements were made. The tissue image was projected onto a charge-coupled device camera (COHU 4815) connected to a videocassette recorder and viewed on a monitor. Measurements were carried out using a 40 × (LUMPFL-WIR, numerical aperture 0.8, Olympus) water immersion objective. The same sites of study were followed throughout the experiment so comparisons could be made directly to baseline levels.

### 2.8. Microhemodynamics

Arteriolar and venular blood flow velocities were measured online by using the photodiode cross-correlation method [31] (Photo Diode/-Velocity Tracker Model 102B, Vista Electronics, San Diego, CA). The measured centerline velocity (V) was corrected according to vessel size to obtain the mean RBC velocity [32]. A video image-shearing method was used to measure vessel diameter (D) [33]. Blood flow (Q) was calculated from the values measured as $Q=\pi xVx{\left(D/2\right)}^{2}$. The data was separated into three groups: arteriole diameter and flow < 60 μm, arteriole diameter and flow > 60 μm, and venule diameter and flow between 20 and 80 μm.

### 2.9. Functional capillary density (FCD)

Functional capillaries, defined as those capillary segments that exhibited RBC transit of at least one RBC in a 45 s period in 10 successive microscopic fields, were assessed, totaling a region of 0.46 mm^2^. Each field had between two and five capillary segments with RBC flow. FCD (cm^−1^), i.e., the total length of RBC perfused capillaries divided by the area of the microscopic field of view, was evaluated by measuring and adding the length of capillaries that exhibited RBC transit in the field of view. The relative change in FCD from baseline levels after each intervention is indicative of the extent of capillary perfusion [34,35].

### 2.10. Pharmacokinetics— exchange transfusion protocol

Pharmacokinetics was studied for 48 h after an infusion with 5 g/dL of ααHb pretreated with the Lactated Ringer’s solution or 5 g/dL of ααHb pretreated with the apoHb-Hp solution. Briefly, C57BL/6 mice were infused into the tail vein at a rate of 100 μL/min tail vein via a syringe pump (Harvard Apparatus, Holliston, MA). Blood samples (70 μL) were taken after infusion at 0, 2, 4, 8, 12, 24, and 48 h, the Hb concentration was then measured via UV–visible spectroscopy.

### 2.11. Organ harvesting and analysis

Following the experimental protocol, blood was collected and centrifuged to separate the plasma. Animals were sacrificed with Fatal Plus (sodium pentobarbital, 300 mg/kg), urine and plasma were collected, and kidneys, liver, spleen, and lungs were harvested. Markers of inflammation, organ function, and organ injury were evaluated. These analyses were performed by the UC San Diego Histology Core via ELISA and flow cytometric analysis of tissue homogenates and plasma. Markers of inflammation, function, and organ injury were evaluated and normalized per gram of tissue using the ELISA kits/assays described in Table S1 Supplementary Table 1.

### 2.12. Statistical analysis

Results are presented as the mean ± standard deviation. Data within each group were analyzed using analysis of variance for repeated measurements (ANOVA, Kruskal-Wallis test). When appropriate, *post hoc* analyses were performed with the Dunn’s multiple comparison test. Comparison between groups was performed using two-way ANOVA (Hb plasma concentration); *post hoc* analyses were performed with Bonferroni posttests. Microhemodynamic data are presented as absolute values and ratios relative to baseline values. A ratio of 1.0 signifies no change from baseline, while lower and higher ratios are indicative of changes proportionally lower and higher than baseline (i.e., 1.5 represents a 50% increase from the baseline level). The same blood vessels and capillary fields were monitored throughout the study, such that direct comparisons to their baseline levels could be performed, allowing for more reliable statistics on small sample populations. All statistics were calculated using computer software (GraphPad Prism 9, GraphPad Software, Inc., San Diego, CA). Changes were considered significant if the p values were less than 0.05. The sample size per experimental group is based on the ANOVA test using an alpha of 0.05 and a power of 0.9 resulting in a minimum acceptable sample size of 15 per group.

## 3. Results

### 3.1. Systemic variables and blood gases

**Fig. 2.**
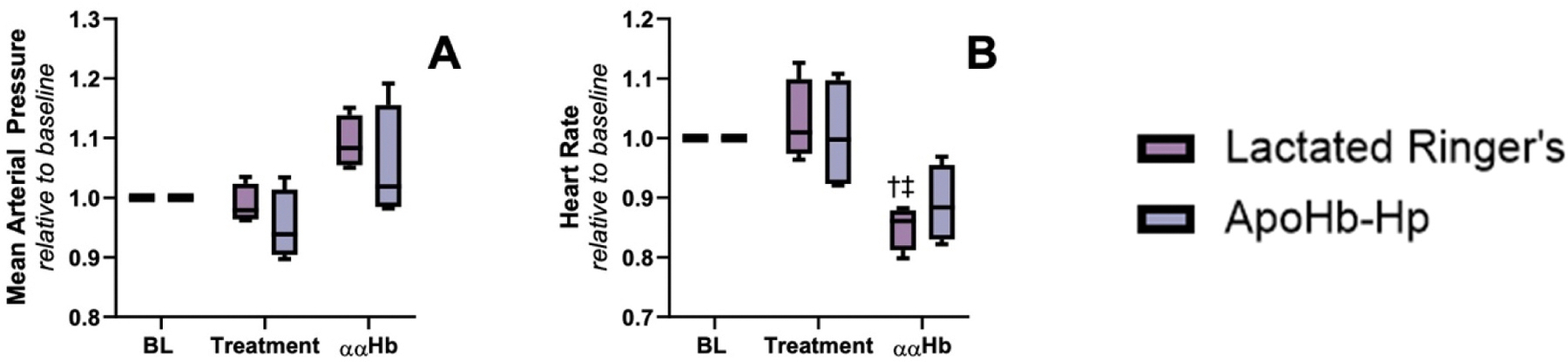
MAP and HR of Golden Syrian hamsters – The Golden Syrian hamsters’ MAP and HR were evaluated during the protocol. The values were normalized to baseline to observe relative changes A) mean arterial pressure and B) heart rate. Each group consisted of 4 hamsters. † vs Baseline, ‡ vs Treatment.

MAP and HR are presented in Fig. 2 and Table 1. Both MAP and HR values reverted to baseline after ααHb challenge with apoHb-Hp pretreatment showing no significance compared to baseline. However, the HR for the lactated Ringer’s control group after ααHb challenge did not return to baseline showing a significant decrease compared to baseline.

Total Hb concentration (tHb), Hct, pH, pO_2_, and pCO_2_ are presented in Table 1. It was observed that the group pretreated with apoHb-Hp reverted tHb, Hct, pH, pO_2_, and pCO_2_ back to baseline levels post ααHb challenge. However, the control group showed a significant decrease in Hct and increase in HR compared to baseline post ααHb challenge.

### 3.2. Pharmacokinetics

**Fig. 3.**
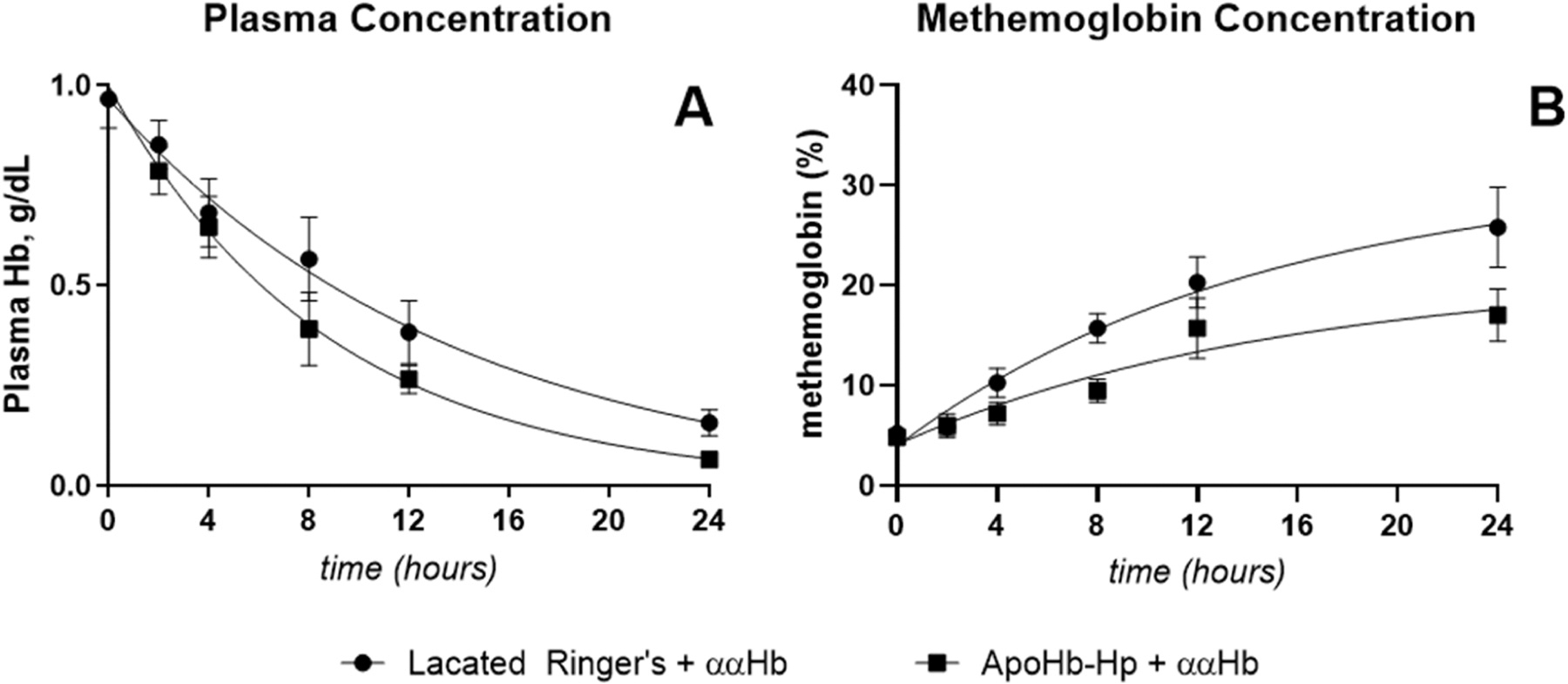
Plasma concentration and methemoglobin concentration. C57BL/6 mice challenged with ααHb have a shorter half-life and slower methemoglobin formation rate when pretreated with apoHb-Hp. A) plasma pharmacokinetics of ααHb with and without the apoHb-Hp complex and B) plasma methemoglobin pharmacokinetics with and without the apoHb-Hp complex.

The plasma kinetics of ferrous [reduced] and ferric (oxidized) ααHb are presented in Fig. 3. Lactated Ringer’s pretreatment resulted in a ααHb half-life of 9.5 h, while apoHb-Hp pretreatment significantly reduced the half-life to 6.1 h. Furthermore, the data also reported that the tau (rate at which 33% of ferrous Hb turns into ferric Hb) of ααHb was equivalent amongst the groups at 14.5 h when pretreated with lactated Ringer’s, and 15.4 h when pretreated with apoHb-Hp. The MAP and HR during these experiments are shown in Supplemental Fig. 1, where pretreatment of apoHb-Hp significantly prevented the hypertensive effects and drop in HR associated with the infusion of ααHb compared to lactated Ringer’s treated animals.

### 3.3. Microvascular hemodynamics

**Fig. 4.**
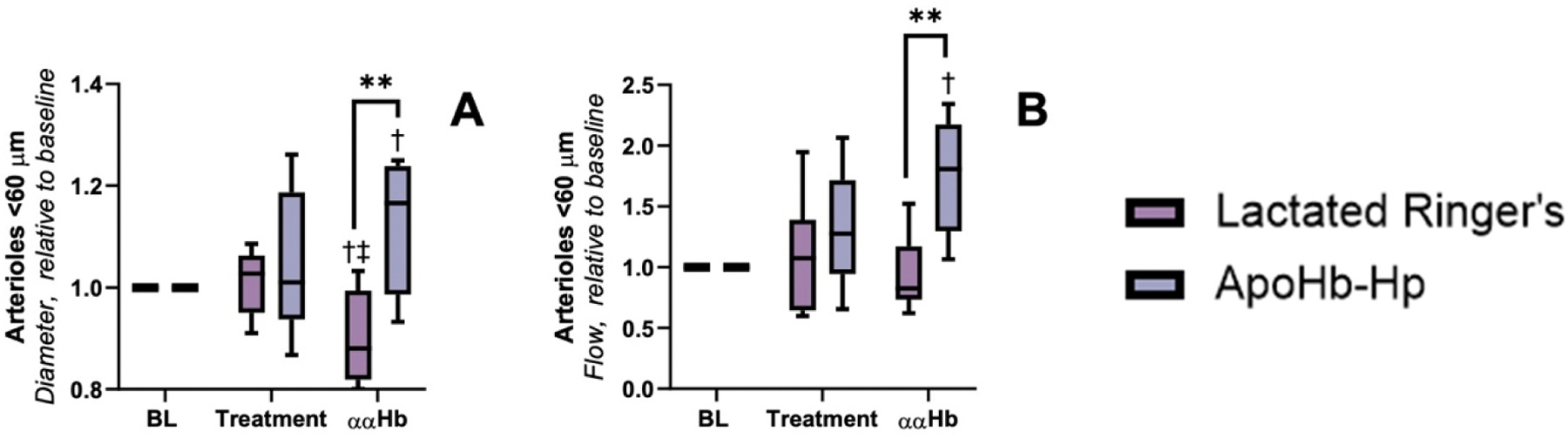
Arterioles < 60 μm. Pretreatment with apoHb-Hp alleviates vasoconstriction and increases microcirculatory blood flow following infusion of ααHb. Relative to baseline measurements were taken to exemplify changes in A) diameter and B) flow of arterioles < 60 μm. † vs *Baseline, ‡* vs *Treatment. P* < 0.005 (**).

**Fig. 5.**
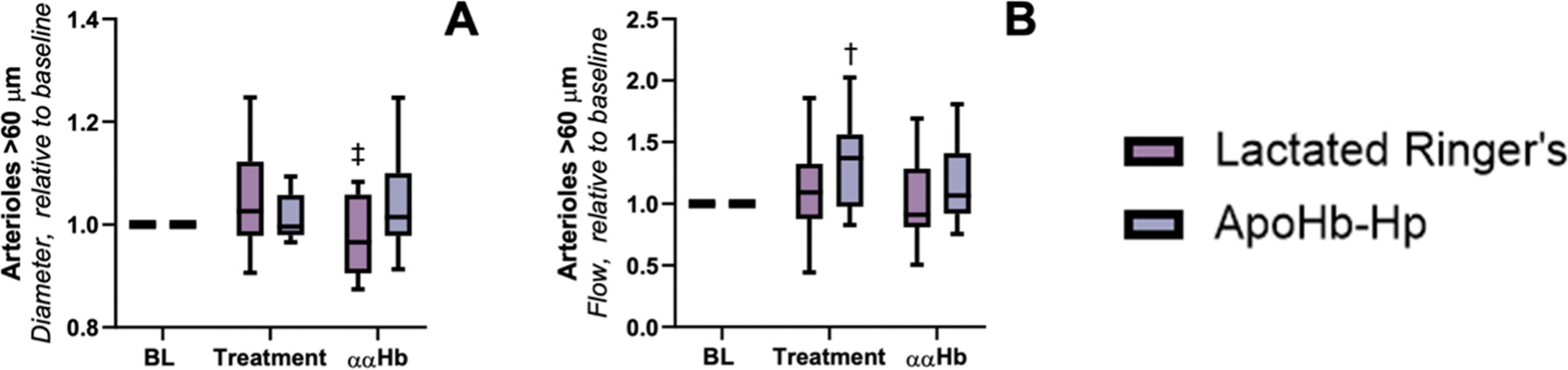
Arterioles > 60 μm. Pretreatment with apoHb-Hp alleviates vasoconstriction and increases microcirculatory blood flow following the infusion of ααHb. Relative to baseline measurements were taken to exemplify changes in A) vessel diameter and B) flow of arterioles > 60 μm. † vs *Baseline, ‡* vs *Treatment*.

**Fig. 6.**
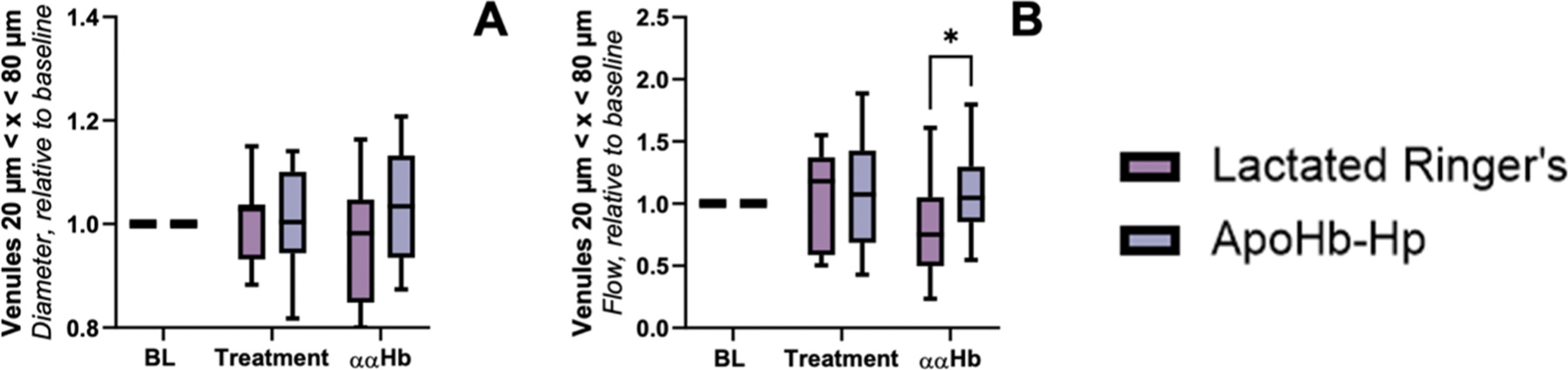
Venules 20 < x < 60 μm. Pretreatment with apoHb-Hp alleviates vasoconstriction and increases microcirculatory blood flow following infusion of ααHb. Relative to baseline measurements were taken to exemplify changes in A) diameter and B) flow of venules 20 < x < 80 μm. † vs *Baseline, ‡* vs *Treatment, P* < 0.05 (*).

Microvascular measurements are presented in Figs. 4–6. The data for this section was segregated by blood vessel diameter for arterioles greater than 60 μm, arterioles less than 60 μm, and venules between 20 and 80 μm. For arterioles < 60 μm, we observed that both pretreatment groups showed no significant difference in arteriole diameter or blood flow compared to baseline. However, post ααHb challenge, the control group showed a significant decrease in arteriole diameter compared to baseline (indicative of vasoconstriction) and the apoHb-Hp group, while the apoHb-Hp group showed a significant increase in arteriole diameter compared to baseline and a significant increase in blood flow compared to the control group. For arterioles > 60 μm, we observe that the arteriole diameter for both groups was unchanged from baseline at pretreatment, but the arteriole diameter of the control group was significantly less than the apoHb-Hp group after ααHb challenge. Also for arterioles > 60 μm, blood flow of the apoHb-Hp pretreatment group was significantly higher than baseline, and both groups were unchanged compared to baseline after ααHb challenge. For venules between 20 and 80 μm, venule diameter for both groups pretreatment and post ααHb challenge were not significantly different compared to baseline. However, blood flow in the control group was significantly less compared to the apoHb-Hp group post ααHb challenge.

### 3.4. Functional capillary density (FCD)

**Fig. 7.**
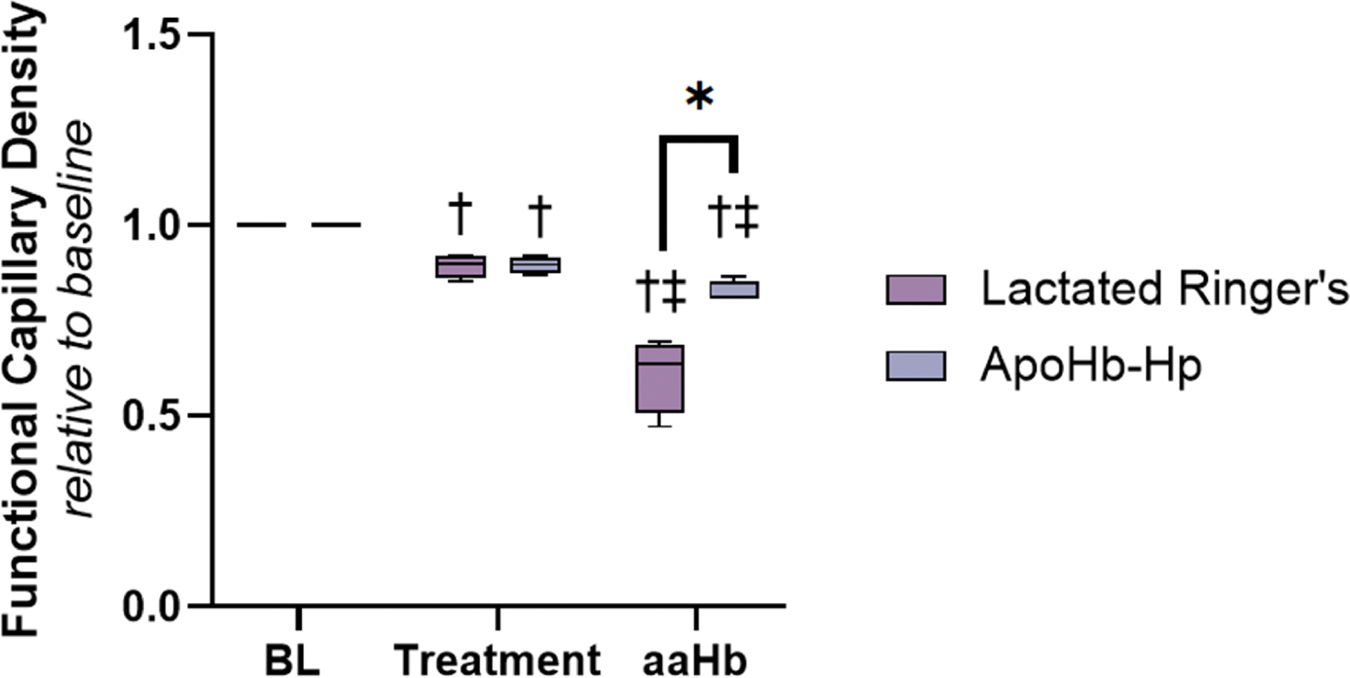
Functional capillary density. Pretreatment with apoHb-Hp significantly reduces the drop in functional capillary density after infusion of ααHb. Relative to baseline measurements were taken to exemplify changes in functional capillaries. † vs *Baseline, ‡* vs *Treatment, P* < 0.05 (*).

FCD normalized to the baseline value is shown in Fig. 7. When comparing the groups to baseline, both the control and the apoHb-Hp groups are significantly lower compared to baseline both during pretreatment and post ααHb challenge. However, the FCD is significantly lower for the control group than the apoHb-Hp group post ααHb challenge, demonstrating better FCD preservation with apoHb-Hp treatment.

### 3.5. Inflammatory markers

**Fig. 8.**
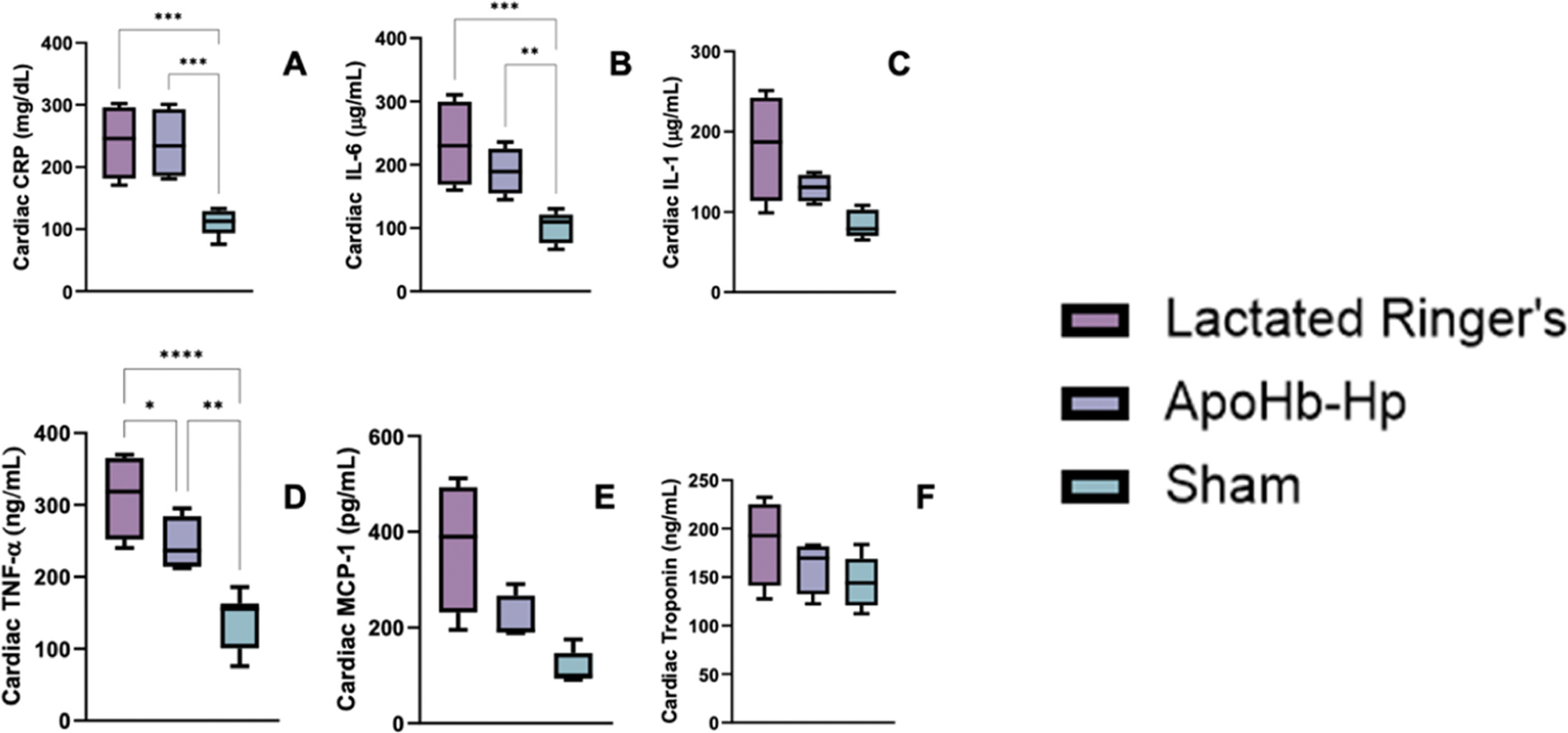
Cardiac inflammation – Pretreatment of apoHb-Hp in Golden Syrian hamsters reduces cardiac inflammation compared to the pretreatment of lactated Ringer’s. The biomarkers measured consisted of A) cardiac c-reactive protein (CRP), B) cardiac IL-6, C) cardiac IL-1, D) cardiac TNF-α, E) cardiac MCP-1, and F) cardiac Troponin. * P < 0.05, ** P < 0.01, *** P < 0.005, **** P < 0.001.

Heart, liver, kidney, spleen, and urine were harvested for all subjects at the end of the protocol. To evaluate inflammation in the heart, cardiac TNF-α, IL-6, IL-1, troponin, CRP, MCP-1 were measured and are presented in Fig. 8. Cardiac TNF-α, IL-6, and CRP showed a significant increase in the control and apoHb-Hp groups post ααHb challenge compared to a sham group (animals that underwent the surgical procedures, but without the experimental protocol). However, for cardiac TNF-α, the apoHb-Hp group was significantly lower that the control group post ααHb challenge. Cardiac troponin, IL-1 and MCP-1 showed no significant difference between the groups post ααHb challenge, however inflammation was still higher in the control group compared to the apoHb-Hp or sham groups post ααHb challenge. All other organ inflammatory data, including kidney, liver, spleen, and urine, which trended towards improved organ function in apoHb-Hp treated animals can be found in Supplementary Figs. 2–7 and the values associated with them are listed in Supplementary Tables 2–8.

## 4. Discussion

The data presented in this study demonstrated the ability of the dual Hb and heme scavenger complex apoHb-Hp to enhance microvascular health and reduce inflammation in vital organs resulting from acellular Hb (ααHb) exposure. This revelation could revolutionize the HBOC field, potentially paving the way for their safer and more widespread clinical application. The infusion of ααHb, while not demonstrating significant effects on systemic parameters when combined with apoHb-Hp, should not be dismissed as harmless. It’s essential to acknowledge that the ααHb dosage used in the current model might have been too conservative to induce ααHb’s notorious hypertensive effects, underscoring the need for higher ααHb dosing to further exacerbate ααHb’s toxicity and subsequent attenuation via apoHb-Hp administration.

When establishing the safety profile of any HBOC, microhemodynamics emerged as a central theme, so we chose to highlight it in this study. ApoHb-Hp was pivotal in maintaining microcirculatory health (i.e. blood vessel diameter, flow, and FCD). Arteriole diameter in the microcirculation is a direct indicator of vasoconstriction. It is induced by ααHb’s ability to extravasate through the blood vessel wall and scavenge NO, a well-documented side-effect associated with the administration of prior generations of small-diameter acellular HBOCs [5,36]. However, when pretreated with apoHb-Hp, ααHb’s vasoactivity was drastically reduced in the arterioles < 60 μm. This observation has profound implications for improving the safety profile of HBOCs. It suggests that Hb and heme scavenger proteins can be used to attenuate the side effects of acellular Hb molecules. The impact of ααHb on microcirculatory blood flow further emphasized the importance of HBOC molecular size in promoting tissue extravasation, suggesting that ααHb’s ability to elicit vasoconstriction might indeed be tied to its molecular dimensions. Previous research has demonstrated the ability of apoHb-Hp to facilitate holoHb-apoHb αβ-dimer exchange and heme-binding [15]. The ability of apoHb-Hp to bind to ααHb was informed by the literature, which supports the ability of ααHb to bind to Hp, however it does so at a much slower rate than ββ crosslinked Hb [37,38]. The increase in molecular size of the resulting Hp- ααHb complex appears to play a pivotal role in preventing vasoactivity. The Hp-ααHb complex is hypothesized to avoid tissue extravasation and limit NO scavenging. This was demonstrated by the lack of vasoconstriction observed in the arterioles. In another study, the apoHb-Hp complex was able to prevent vasoconstriction, systemic hypertension, and oxidative tissue injury associated with low molecular weight polymerized human Hb [39].

Moreover, the implications of the observed shorter half-life of ααHb in circulation when combined with apoHb-Hp hold profound clinical significance. This could be a pivotal factor in augmenting their therapeutic potential. HBOCs auto-oxidize in circulation, and the accelerated elimination via apoHb-Hp could potentially prevent the accumulation of HBOC oxidative damage. However, the reduced half-life of the Hp-ααHb complex could still serve as a therapeutic window to extend the “Golden Hour”. The “Golden Hour” is a concept centered around the idea that a patient will survive a severe trauma if transported to a medical facility for treatment within one hour of injury [40]. The Hp-ααHb complex will provide immediate benefit to hypoxic tissues, thus extending the golden hour as tissue necrosis due to hypoxia is limited.

Beyond these revelations, the administration of apoHb-Hp introduces a new dimension to the study’s significance. By augmenting heme-binding capacity within the bloodstream, apoHb-Hp presents a unique avenue for mitigating vasoactivity and reducing the concentration of inflammatory cytokines in the circulation, typically associated with free heme [18,19]. This discovery’s implications extend to the innate immune system, where heme often triggers responses that can exacerbate existing medical conditions [19,20].

Lastly, the apoHb-Hp complex significantly improves FCD, a key indicator of microcirculatory health. The subsequent increase in hydrostatic pressure at the arteriole end of the capillaries induces a more significant number of functional and denser capillary beds, a physiological state inherently advantageous for tissue oxygenation and overall health. The marked disparity in FCD, when compared to ααHb’s effects, underscores the immense potential of the apoHb-Hp complex in mitigating the adverse outcomes associated with acellular HBOCs. These findings strongly suggest that apoHb-Hp could be the missing piece in the HBOC puzzle. The notable reduction in inflammation observed in vital organs, including the heart, spleen, liver, kidney, and plasma, cannot be overstated. This discovery alone harbors tremendous promise for the future of blood transfusion therapies, as inflammation remains a common adverse effect capable of significantly impacting patient outcomes.

In conclusion, this study clarifies the mechanism of ααHb side-effects, and the information is important for the development of HBOCs. However, this does mark a significant leap forward in pursuing safer and more effective HBOCs as we demonstrated that the apoHb-Hp complex has the potential to markedly reduce HBOC-associated toxicities.

## Supplementary Material

supplemental

Appendix A. Supporting information

Supplementary data associated with this article can be found in the online version at doi:10.1016/j.biopha.2024.116569.

## Figures and Tables

**Table 1:** Laboratory parameters. Total Hb concentration, Hct, MAP, HR, pH, pCO_2_, and pO_2_ were measured at each phase of the experiment. The values are shown as the mean ± standard deviation with † to distinguish a significant change from baseline.

	Baseline	Lactated Ringer’s	ααHb	Baseline	apoHb-Hp	ααHb

**tHb [g/dL]**	16 ± 1	15 ± 1	15 ± 1	16 ± 1	15 ± 1	15 ± 1
**Hct [%]**	49 ± 2	48 ± 3	46 ± 3^†^	49 ± 1	46 ± 1	45 ± 3
**MAP [mmHg]**	118 ± 8	116 ± 6	129 ± 13	111 ± 12	105 ± 9	117 ± 14
**HR [bpm]**	462 ± 30	474 ± 19	393 ± 30^†^	442 ± 19	443 ± 24	393 ± 29
**pH**	7.36 ± 0.03	7.37 ± 0.04	7.34 ± 0.02	7.35 ± 0.03	7.35 ± 0.04	7.34 ± 0.01
**pCO_2_ [mmHg]**	54.8 ± 4.6	51.7 ± 6.0	49.8 ± 4.7	59.1 ± 6.2	55.2 ± 2.6	53.4 ± 3.0
**pO_2_ [mmHg]**	59.1 ± 9.7	69.1 ± 14.2	69.4 ± 19.4	68.0 ± 15.1	68.2 ± 4.5	66.4 ± 12.1

† vs Baseline.

## Data Availability

Data will be made available on request.
