# Supplementary material for: Toxic side-effects of diaspirin cross-linked human hemoglobin are attenuated by the apohemoglobin-haptoglobin complex
Source: Biomed Pharmacother. Author manuscript; Available in PMC 2026 Jan 19. (PMC12814934; doi:10.1016/j.biopha.2024.116569)
Supplement: supplemental [file NIHMS2131301-supplement-supplemental.docx]

**Supplementary Material**

| **Method** | **Kit/Assay** | **Analyte** | **Vendor** |
| --- | --- | --- | --- |
| ELISA | KA1625 | AST | Abnova Corp, Taiwan |
| ELISA | KA4189 | ALT | Abnova Corp, Taiwan |
| ELISA | BMS625 | IL-6 | Thermo Fisher, Waltham, MA |
| ELISA | BMS629 | IL-10 | Thermo Fisher, Waltham, MA |
| ELISA | KB02-H2 | Creatinine | Arbor Assays Inc, Ann Arbor, MI |
| ELISA | K024-H5 | BUN | Arbor Assays Inc, Ann Arbor, MI |
| ELISA | ERCXCL1 | CXCL-1 | Thermo Fisher, Waltham, MA |
| ELISA | MCA-155 | Ferritin | Serotec, Oxford, UK |
| ELISA | BA-E-6600 | Catecholamines | ImmunoSmol, France |
| ELISA | ERLCN2 | Urine NGAL | Thermo Fisher, Waltham, MA |
| ELISA | ab235627 | Bilirubin | Abcam, Cambridge, UK |
| Luminex technology/ Miliplex Immunoassay | RECYTNMAG-65K | MCP-1 | Millipore Corporation, Massachusetts, USA |
| Luminex technology/ Miliplex Immunoassay | RECYTNMAG-65K | TNF-α | Millipore Corporation, Massachusetts, USA |
| ELISA | ab246529 | Cardiac Troponin | Abcam, Cambridge, UK |
| ELISA | Ab256398 | CRP | Abcam, Cambridge, UK |
| ELISA | Ab108797 | ANP | Abcam, Cambridge, UK |

**Supplemental Table 1 ELISA Kits.** These are the ELISA kits used to determine organ function and inflammation.


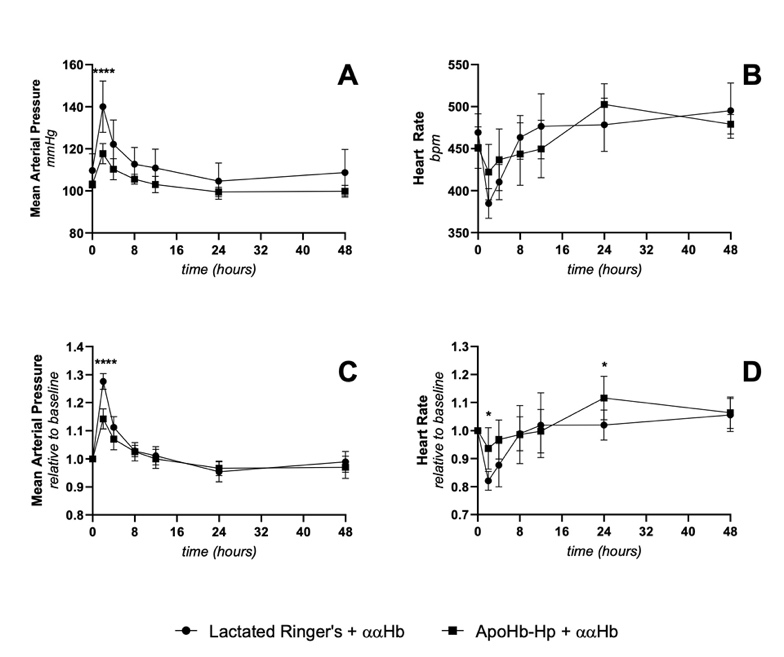


**Supplemental Figure 1 MAP and HR of C57BL/6 Mice** ApoHb-Hp pretreatment prevents hypertension and reduces heart rate after infusion of ααHb. A] Mean arterial pressure [MAP] and B] Heart rate [HR] after ααHb challenge with either pretreatment with lactated Ringer’s or apoHb-Hp. Relative to baseline measurements of C] MAP and D] HR following ααHb challenge pretreated with either lactated Ringer’s or apoHb-Hp. Compared to baseline, P<0.05 [*], P<0.0001[****]


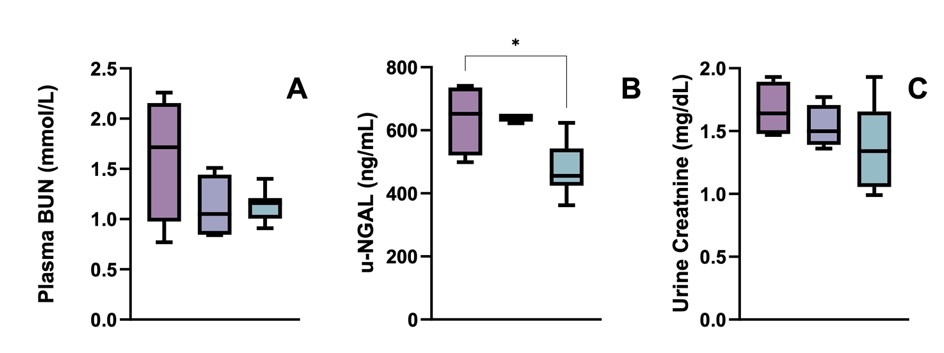


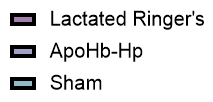


**Supplemental Figure 2 Kidney Function.** Pretreatment with apoHb-Hp improves kidney function after infusion of ααHb. Biomarkers of kidney injury A] Plasma BUN, B] urine-NGAL, and C] urine creatinine were measured to determine any acute kidney damage induced by ααHb. * P < 0.05


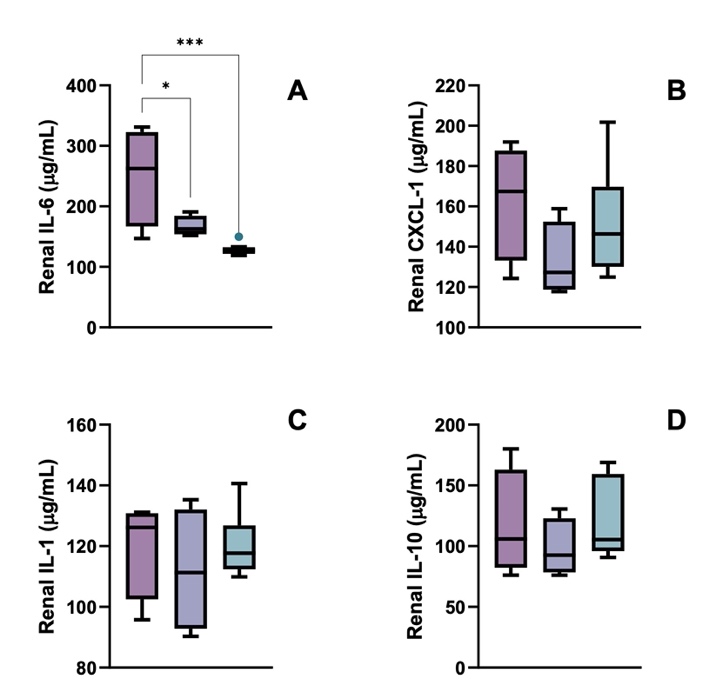

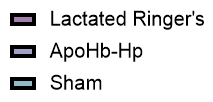


**Supplemental Figure 3 Kidney Inflammation.** Pretreatment with apoHb-Hp improves kidney inflammation after infusion of ααHb. Biomarkers of kidney inflammation: A] Renal IL-6, B] Renal CXCL-1, C] Renal IL-1, and D] Renal IL-10 were measured to determine acute inflammation induced by ααHb. * P < 0.05, *** P < 0.005


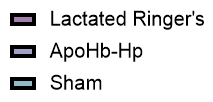

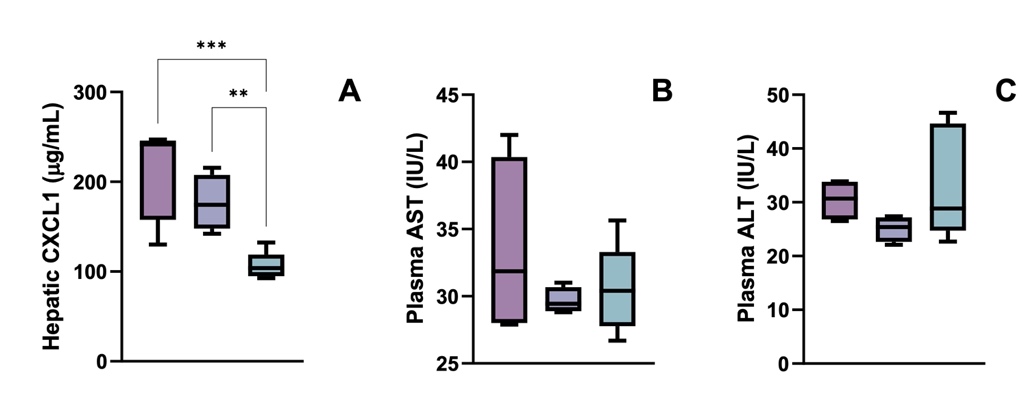


**Supplemental Figure 4 Liver Inflammation and Function** Pretreatment with apoHb-Hp improves liver function and reduces liver inflammation after infusion of ααHb. Biomarkers of liver inflammation and function: A] Hepatic CXCL-1, B] Plasma AST, and C] Plasma ALT were measured to determine acute inflammation in the liver and liver function induced by the ααHb challenge pretreated with the apoHb-Hp complex or control. ** P < 0.01, *** P < 0.005


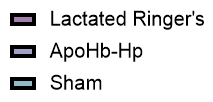

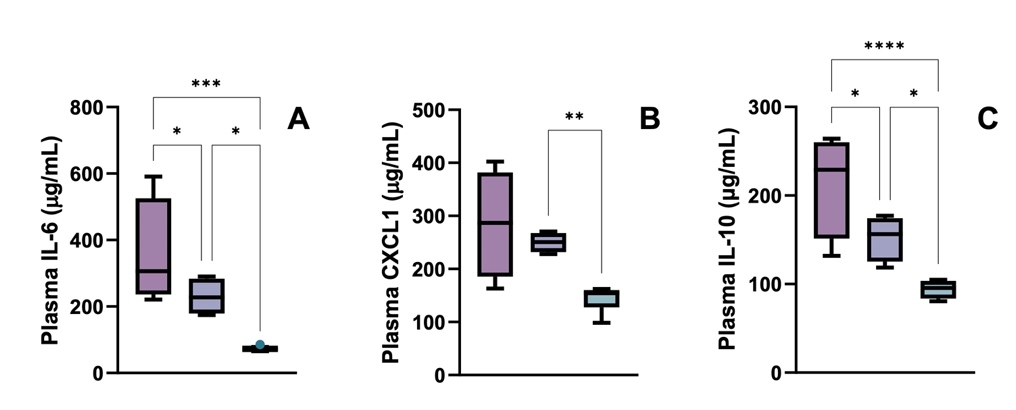


**Supplemental Figure 5 Inflammatory Cytokines in the Plasma**. Pretreatment with apoHb-Hp reduces inflammatory cytokines in the plasma after infusion of ααHb. Biomarkers of inflammatory cytokines in the plasma: A] Plasma IL-6, B] Plasma CXCL-1, and C] Plasma IL-10 were measured to determine acute vascular inflammation induced by ααHb. * P < 0.05, ** P < 0.01, *** P < 0.005, **** P < 0.001


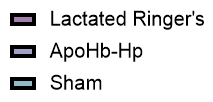

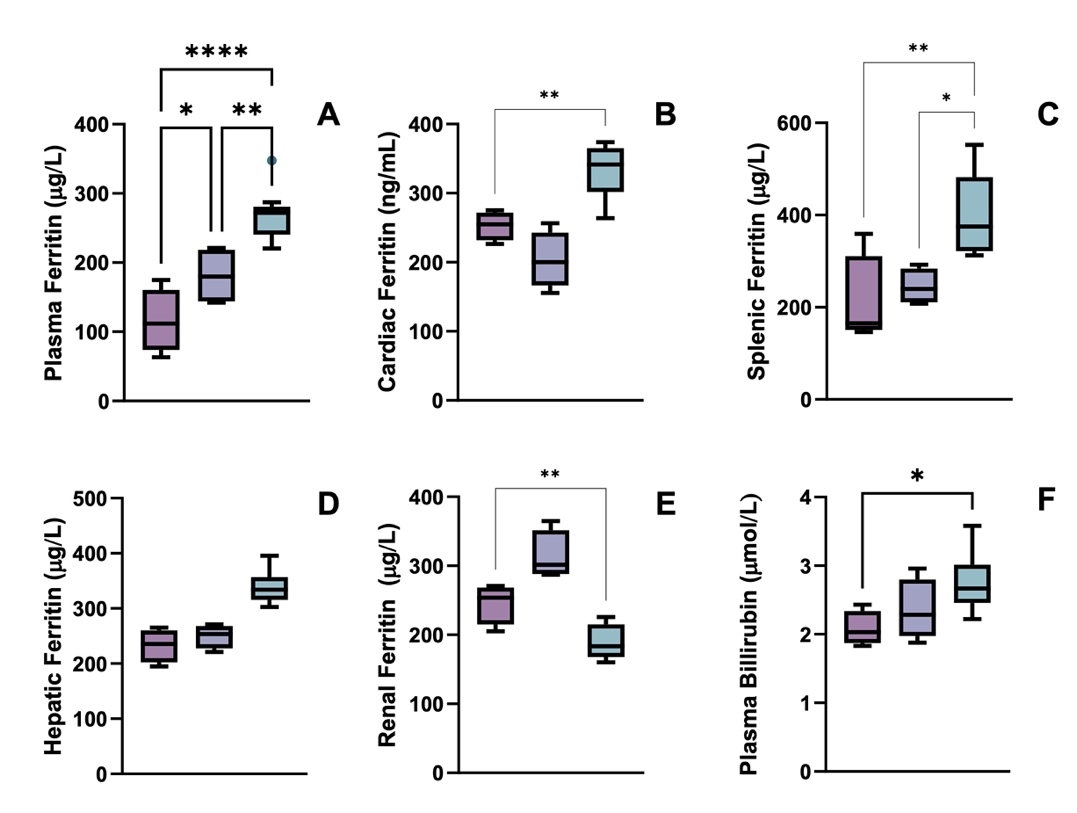


**Supplemental Figure 6 Ferritin and Bilirubin Concentrations**. Pretreatment with apoHb-Hp increases ferritin levels and maintains bilirubin concentration in the plasma after infusion of ααHb. Biomarkers of iron transport: A] Plasma ferritin, B] Cardiac ferritin, C] Splenic ferritin, D] Hepatic ferritin, E] Renal ferritin, and F] Plasma bilirubin were measured to determine iron transport induced by infusion of ααHb. * P < 0.05, ** P < 0.01, *** P < 0.005, **** P < 0.001


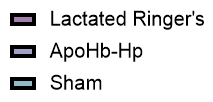

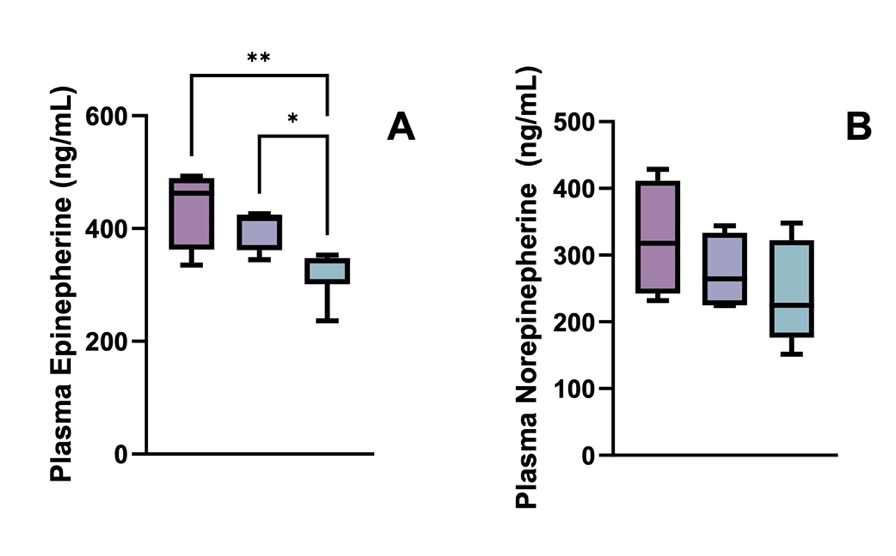


**Supplemental Figure 7 Catecholamines**. Pretreatment with apoHb-Hp reduces catecholamine concentrations after infusion of ααHb. A] Plasma epinephrine, B] Plasma norepinephrine were measured to determine central nervous system reactivity induced by the ααHb challenge pretreated with the apoHb-Hp complex or control. * P < 0.05, ** P < 0.01, *** P < 0.005, **** P < 0.001

| **20 Minutes After Hypovolemic Infusion** | | | |
| --- | --- | --- | --- |
|  | *Control + ααHb* | *ApoHb-Hp + ααHb* | *Sham* |
| **Cardiac CRP (mg/dL)** | 241.1 ± 60.0^†^ | 237.6 ± 57.1^†^ | 110.2 ± 20.3 |
| **Cardiac IL-6 (µg/mL)** | 232.8 ± 68.3^†^ | 189.7 ± 37.3^†^ | 102.7 ± 23.8 |
| **Cardiac IL-1 (µg/mL)** | 181.0 ± 66.8^†‡^ | 130.1 ± 17.0^†^ | 84.0 ± 16.3 |
| **Cardiac TNF-a (ng/mL)** | 311.8 ± 59.6 | 245.0 ± 37.5 | 138.0 ± 37.5 |
| **Cardiac MCP-1 (pg/mL)** | 371.6 ± 136.8 | 216.8 ± 49.3 | 118.0 ± 31.0 |
| **Cardiac Troponin (ng/mL)** | 186.4 ± 44.3 | 161.3 ± 27.3 | 144.7 ± 25.3 |
| † versus sham,  ‡ versus *ApoHb-Hp + ααHb* | | | |

**Supplemental Table 2 Cardiac Inflammation.** Measurements of cardiac CRP, cardiac IL-6, cardiac IL-10, cardiac TNF-α, cardiac MCP-1 and cardiac troponin are indicative of cardiac function. Three Golden Syrian hamsters were sacrificed without undergoing the experimental protocol to establish a sham group.

Significant compared to sham [†]; significant compared to ApoHb-Hp + ααHb [‡].

| **20 Minutes After Hypovolemic Infusion** | | | |
| --- | --- | --- | --- |
|  | *Control + ααHb* | *ApoHb-Hp + ααHb* | *Sham* |
| **Plasma BUN (mmol/L)** | 1.6 ± 0.6 | 1.1 ± 0.3 | 1.1 ± 0.1 |
| **Urine NAGL (ng/mL)** | 636.2 ± 114.7^†^ | 641.3 ± 13.0 | 476.8 ± 80.9 |
| **Urine Creatinine (mg/dL)** | 1.7 ± 0.2 | 1.5 ± 0.2 | 1.3 ± 0.3^†^ |
| † versus sham,  ‡ versus *ApoHb-Hp + ααHb* | | | |

**Supplemental Table 3 Kidney Function.** Measurements of plasma blood urea nitrogen [BUN], urine neutrophil gelatinase-associated lipocalin [NAGL], and urine creatinine were measured to evaluate kidney function. Three Golden Syrian hamsters were sacrificed without undergoing the experimental protocol to establish a sham group.

Significant compared to sham [†]; significant compared to ApoHb-Hp + ααHb [‡].

| **20 Minutes After Hypovolemic Infusion** | | | |
| --- | --- | --- | --- |
|  | *Control + ααHb* | *ApoHb-Hp + ααHb* | *Sham* |
| **Renal IL-6 (µg/mL)** | 250.7 ± 81.8^†^ | 167.1 ± 16.8^†^ | 128.5 ± 9.5 |
| **Renal IL-1 (µg/mL)** | 119.8 ± 16.5 | 112.1 ± 20.4 | 120.5 ± 10.1 |
| **Renal CXCL-1 (µg/mL)** | 162.7 ± 28.8 | 132.8 ± 18.5 | 150.8 ± 26.7 |
| **Renal IL-10 (µg/mL)** | 117.0 ± 44.5 | 98.0 ± 23.8 | 120.8 ± 32.1 |
| † versus sham,  ‡ versus *ApoHb-Hp + ααHb* | | | |

**Supplemental Table 4 Kidney Inflammation.** Measurements of renal IL-6, renal IL-1, renal CXCL-1 and renal IL-10 were used to quantify kidney inflammation. Three Golden Syrian hamsters were sacrificed without undergoing the experimental protocol to establish a sham group.

Significant compared to sham [†]; significant compared to ApoHb-Hp + ααHb [‡].

| **20 Minutes After Hypovolemic Infusion** | | | |
| --- | --- | --- | --- |
|  | *Control + ααHb* | *ApoHb-Hp + ααHb* | *Sham* |
| **Hepatic CXCL-1 (µg/mL)** | 215.1 ± 56.8^†^ | 176.6 ± 31.1^†^ | 107.5 ± 14.2 |
| **Plasma AST (IU/L)** | 33.4 ± 6.7 | 29.7 ± 1.0 | 30.4 ± 3.2 |
| **Plasma ALT (IU/L)** | 30.5 ± 3.8 | 25.1 ± 2.4 | 33.2 ± 2.1 |
| † versus sham,  ‡ versus *ApoHb-Hp + ααHb* | | | |

**Supplemental Table 5 Liver Inflammation and Function.** Measurements of hepatic chemokine ligand 1 [CXCL-1], plasma aspartate transaminase [AST], and plasma alanine transaminase [ALT] were used to quantify inflammation and liver function. Three Golden Syrian hamsters were sacrificed without undergoing the experimental protocol to establish a sham group.

Significant compared to sham [†]; significant compared to ApoHb-Hp + ααHb [‡].

| **20 Minutes After Hypovolemic Infusion** | | | |
| --- | --- | --- | --- |
|  | *Control + ααHb* | *ApoHb-Hp + ααHb* | *Sham* |
| **Plasma IL-6 (µg/mL)** | 356.3 ± 162.8^†‡^ | 230.0 ± 55.6^†^ | 73.0 ± 5.7 |
| **Plasma CXCL-1 (µg/mL)** | 284.9 ± 101.4 | 250.0 ± 18.5^†^ | 144.9 ± 22.2 |
| **Plasma IL-10 (µg/mL)** | 213.6 ± 59.0^†‡^ | 152.0 ± 25.8^†^ | 94.63 ± 9.8 |
| † versus sham,  ‡ versus *ApoHb-Hp + ααHb* | | | |

**Supplemental Table 6 Vascular Inflammation** Measurements of plasma interleukin-6 [IL-6], plasma chemokine ligand 1 [CXCL1], and plasma interleukin-10 [IL-10] were used to quantify vascular inflammation. Three Golden Syrian hamsters were sacrificed without undergoing the experimental protocol to establish a sham group.

Significant compared to sham [†]; significant compared to ApoHb-Hp + ααHb [‡].

| **20 Minutes After Hypovolemic Infusion** | | | |
| --- | --- | --- | --- |
|  | *Control + ααHb* | *ApoHb-Hp + ααHb* | *Sham* |
| **Plasma Ferritin (μg/L)** | 269.3 ± 37.3^†‡^ | 180.6 ± 40.9^†^ | 269.3 ± 37.3 |
| **Cardiac Ferritin (ng/L)** | 203.1 ± 41.3^†^ | 252.8 ± 20.9 | 333.4 ± 37.5 |
| **Splenic Ferritin (μg/L)** | 208.9 ± 100.5^†^ | 244.8 ± 38.3^†^ | 399.9 ± 86.6 |
| **Hepatic Ferritin (μg/L)** | 232.7 ± 30.2 | 249.8 ± 21.4 | 339.0 ± 28.7 |
| **Renal Ferritin (μg/L)** | 245.9 ± 29.1^†^ | 313.7 ± 35.8 | 189.1 ± 24.6 |
| **Plasma Bilirubin (μmol/L)** | 2.1 ± 0.3^†^ | 2.4 ± 0.4 | 2.8 ± 0.4 |
| † versus sham,  ‡ versus *ApoHb-Hp + ααHb* | | | |

**Supplemental Table 7 Ferritin and Bilirubin Concentrations** Measurements of plasma ferritin, cardiac ferritin, splenic ferritin, hepatic ferritin, renal ferritin, and plasma bilirubin were used to quantify levels of iron. Three Golden Syrian hamsters were sacrificed without undergoing the experimental protocol to establish a sham group.

Significant compared to sham [†]; significant compared to ApoHb-Hp + ααHb [‡].

| **20 Minutes After Injection** | | | |
| --- | --- | --- | --- |
|  | *Control+ ααHb* | *ApoHb-Hp + ααHb* | *Sham* |
| **Plasma Epinephrine (ng/mL)** | 438.2 ± 71.5^†^ | 401.4 ± 38.2^†^ | 320.8 ± 37.99 |
| **Plasma Norepinephrine (ng/mL)** | 323.9 ± 88.1 | 274.3 ± 59.3 | 241.4 ± 73.4 |
| † versus sham,  ‡ versus *ApoHb-Hp + ααHb* | | | |

**Supplemental Table 8 Catecholamines** Measurements of plasma epinephrine and plasma norepinephrine. Three Golden Syrian hamsters were sacrificed without undergoing the experimental protocol to establish a sham group.

Significant compared to sham [†]; significant compared to ApoHb-Hp + ααHb [‡].
